# Perceptions of Speed and Risk: Experimental Studies of Road Crossing by Older People

**DOI:** 10.1371/journal.pone.0152617

**Published:** 2016-04-07

**Authors:** Annie A. Butler, Stephen R. Lord, Richard C. Fitzpatrick

**Affiliations:** 1 Neuroscience Research Australia, Sydney, Australia; 2 University of New South Wales, Sydney, Australia; Beihang University, CHINA

## Abstract

Crossing a road safely is a complex task requiring good sensorimotor function and integration of information about traffic speed, distances and one’s own speed. Poor judgement through age-related sensorimotor or cognitive impairment or a predisposition to take risks could lead to errors with serious consequences. On a simulated road, 85 participants (age ≥70 years) were asked to cross in front of an approaching car with a clearance as small as considered safe in two conditions; (1) with nothing else to attend to (*free crossing*) and (2) with an additional ball-gathering task while waiting to cross (*task crossing*). Participants were categorised according to their crossing outcome (failed to cross, ‘hit’, exact, safe, cautious). Participants also performed two sub-studies; (1) the perception of the time-to-arrival of moving objects and (2) the perception of own gait speed. Physical and cognitive function and everyday risk-taking behaviour were also assessed. In free crossing, clearances varied but no participants were “hit” by the car. In task crossing, participants allowed smaller clearances and 10% of participants would have been hit while 13% missed the opportunity to cross altogether. Across a wide range of physical and cognitive measures, including perceived and actual gait speed, a consistent pattern was observed in the task crossing condition. The *exact* group performed best, the ‘*hit’*, *safe* and *cautious* groups performed less well while those who missed the opportunity (*fail*) performed worst. The *exact* group reported taking the greatest risks in everyday life whereas the remaining groups reported being cautious. In conclusion, we found older people with poorer perceptual, physical and cognitive function made inappropriate and risky decisions in a divided attention road-crossing task despite self-reports of cautious behaviour in everyday life.

## Introduction

Crossing a road in traffic can be a complex task but has become an essential skill in urban life. It requires a combination of immediate decisions and longer-term judgements of the movements of vehicles and one’s own mobility. Pedestrian injuries are a particular problem for older people. People over 70 years make up 10% of the population in New South Wales, Australia but account for up to one-third of pedestrian fatalities [1] and are more likely to suffer serious injuries than younger adults [2]. The great majority of collisions occur while older people are crossing suburban roads in daylight and fault is infrequently attributed to motorist actions [3, 4], implying that older pedestrians are making errors of judgement.

Successful crossing relies on integrating perception and action [5, 6] in that vehicles coming from different directions must be identified, usually by sight and sound and their times to arrival must be estimated, most likely through visual signals of optic flow [7]. Pedestrians must also allow for an appropriate safety margin to cross before the approach of oncoming vehicles, i.e. make a prediction about locomotor trajectory using prior knowledge and sensory information from different sources. These sources include visual signals of self-motion [7–11], vestibular signals [12, 13] and podokinetic somatosensory signals [14]. After initiating a crossing, it may be necessary to continue observing oncoming vehicles, update the estimates of their time to arrival, and adjust walking speed and trajectory as required. In fact, models of pedestrian behaviour and flow identify complex interactions among all road users [15, 16].

Declines in function are documented across every physiological domain central to locomotor control: visual, auditory, somatosensory, vestibular, muscle strength, muscle contraction speed, reaction time, central motor function and higher cognitive function [17–20]. For some older people, unforeseen risks in crossing decisions may be due to unnoticed age-related declines in performance. For others, however, a tendency towards risk-taking irrespective of perceived ability might lead to unsafe decisions. Several studies of road-crossing decisions, all in virtual road-crossing environments, have shown that older people make more dangerous decisions than young people, particularly in complex traffic environments [21–28], and that these decisions are associated with declines in physical and cognitive function [22, 27, 29, 30]. Few of the above studies has investigated associations between road crossing decisions and physical and cognitive function in an ecological design that involved participants actually crossing in front of ‘virtual’ traffic [27, 30]. These studies investigated only one test of physical ability, i.e. gait, and did not assess whether road-crossing decisions were influenced by the ability to perform dual tasks or switch tasks.

In our main study, we investigated road crossing behaviours in older participants by using a physical road and vehicle scaled down in distance and speed in a controlled laboratory environment. Two sub-studies investigated perceptions of walking speed and perceptions of the time to arrival of moving objects. The main aims were to determine the effect of an additional task on crossing decisions and to identify perceptual, physical and cognitive factors that contribute to safe and unsafe crossing behaviour. Specific hypotheses were: (i) misjudgements of the speed of moving objects and own gait speed would influence road-crossing behaviour, (ii) reduced physical and cognitive function would be associated with more unsafe crossing decisions (particularly in a condition requiring an additional task) and (iii) reported risk taking in everyday life would be associated with riskier road crossing decisions in the laboratory.

## Methods

### Participants

The sample comprised 85 people (40 men, 45 women) aged 70–90 years (mean 78.0 ± 5.0 SD) without neurological, cardiovascular or major musculoskeletal impairments who were drawn from a larger study of fall risk factors in older people who were recruited by random selection from the State Electoral Rolls of Sydney’s eastern suburbs [31]. All lived in private households and scored 24 or more on the Mini Mental State Examination (mean 27.5 ± 1.7 SD). Participants gave written informed consent, and the experimental procedures were in accordance with the Declaration of Helsinki and approved by the University of New South Wales Human Research Ethics Committee (HC05224).

### Experimental Setup and protocol

#### Study 1—Road crossing

**Fig 1:**
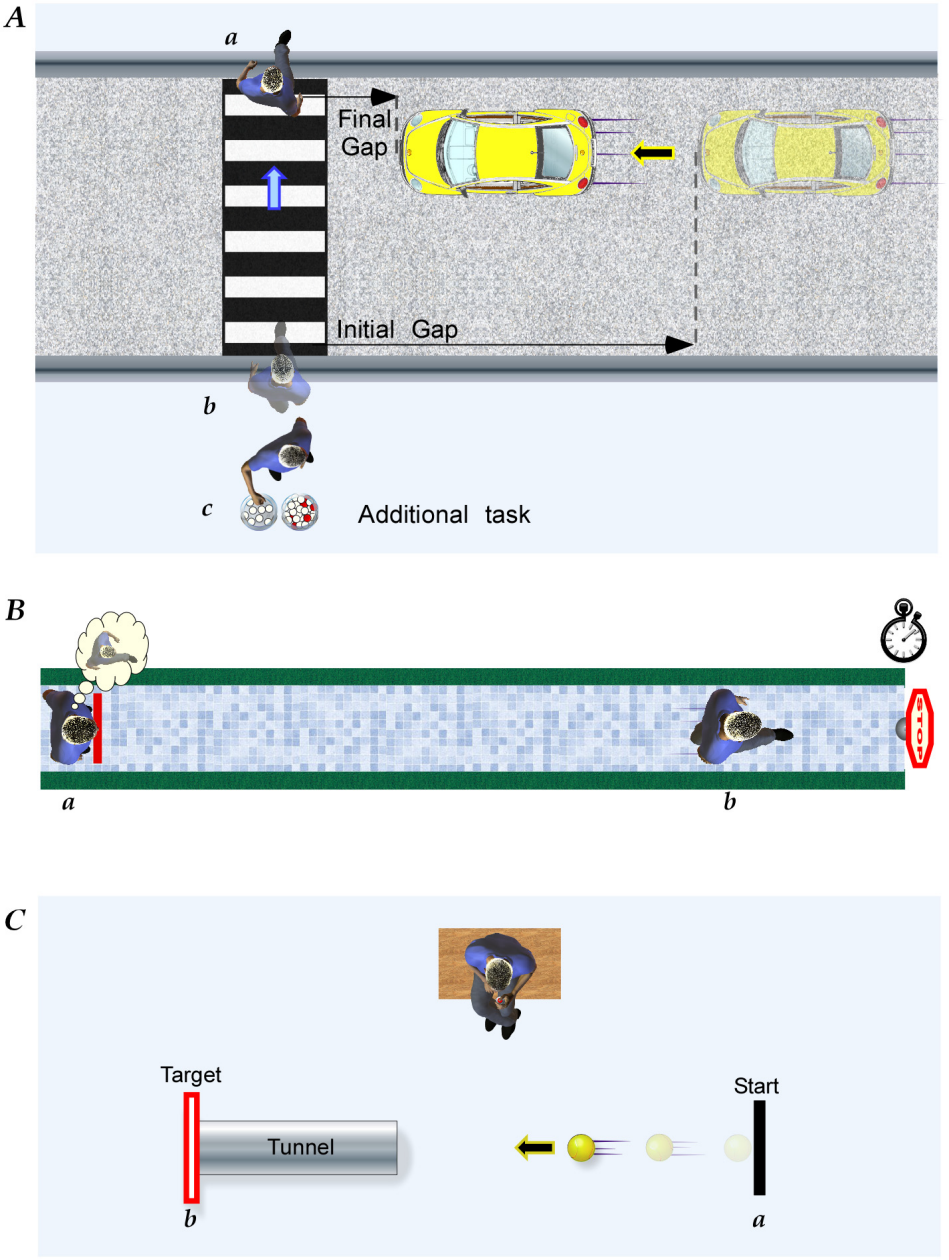
Methods. A. Study 1: Road Crossing. After crossing the road (*a*→*b*), participants waited, either at the road edge (*free crossing*) or performing the ball-gathering task (*task crossing*) at *c*. On the return, crossing in front of the oncoming car (*b*→*a*), the initial and final gaps to the car were measured. B. Study 2: Perceived time to walk to a target. Participants stood at the end of a 5m or 10m path (*a*) and imagined walking to the end, and indicated when they arrived. They then walked the paths (*b*) and the actual time taken was measured. C. Study 3: Perceived time-to-arrival of a moving object. Projected onto the floor was a virtual “ball” that left the start line (*a*) and moved towards a target (*b*) at constant speed. Before reaching the target it disappeared into a tunnel. Participants indicated with a finger press the time they estimated the ball would reach the target.

A simulated road (10m long × 4.2m wide) and a marked pedestrian crossing (0.67 m wide) were created in a well-lit open laboratory (10 m × 6 m; Fig 1A). A mock-up Styrofoam and aluminium car (1.6×1.3×1.2m: l×w×h) travelled back and forth along the road, driven by a motor and cable under computer control (*i*.*e*. a cable car). From a stationary start at one end of the road, the car accelerated to its maximum speed (0.7ms^-1^) within 2.8m, remained at this speed along most of the road, and then decelerated with the opposite profile to stop momentarily at the far end of the road before making the reverse journey. The round trip took 43s. This movement of the car and distances to be walked allowed all participants sufficient time to complete the crossing safely. The car could be stopped abruptly at any time by the experimenter. Two protocols, *free crossing* and *task crossing*, were conducted in a random order. Later analysis did not indicate a significant effect of order on outcome measures.

For the *free crossing* protocol, participants stood at the roadside at the pedestrian crossing and waited for the car to pass before crossing to the other side. They then waited and observed the car as it returned back towards them. They were asked to cross in front of the car leaving the shortest possible crossing gap that they considered safe.

For the *task crossing* protocol, as with the free crossing, participants crossed to the other side, waited and crossed back in front of the oncoming car as it returned. While waiting, however, they had to move as many white balls (5cm diameter) as possible from a jar of mixed red and white balls into another container using one hand. Participants faced away from the road while doing this but could easily turn to view the approaching car. This task was designed to be easy but compelling and the inclusion of red balls divided concentration and visual attention between the task and the car. The instruction given was to obtain as many white balls as possible but cross back across the road safely (without being hit). To determine the extent to which attention was divided, the time taken to gather the same number of balls was later measured without the distraction of the car.

A Codamotion motion capture system (Charnwood Dynamics Ltd.) recording 3-D data at 50 Hz was used to measure the positions of the participant and the car throughout the experiment. Active markers were placed over the fibula heads and acromion processes bilaterally, and on the front outer corners (bumpers) of the car. The distance between the car and participant as (i) the participant initiated the first step onto the road (*Initial Gap*) and (ii) as the participant was in line with the far side of the car and about to leave its path (*Final Gap*) were recorded, as were walking speed and time to cross the road. A customised Labview program was used to calculate these measures from the data collected with the Codamotion system.

Crossings were categorised as: *fail* (the participant left it too late and had to turn back to avoid being hit), *hit* (experimenter stopped the car to avoid collision), *exact* (participant crossed safely with a narrow margin < 0.5 m), *safe* (participant crossed safely with a margin of 0.5m–1.2m) and *cautious* (participants crossed safely with a margin of>1.2m). These categories were selected based on the average walking speed of older people (1.0ms^-1^), [32], and the speed of the car (0.7ms^-1^). An *exact* gap of <0.5m equated to less than one second of clearance between the car and the pedestrian. A safe gap of between 0.7 m and 1.2 m allowed an additional 1 second gap.

#### Study 2—Perceived time to walk to a target

Participants (N = 75) stood before a straight 5m path in an open flat courtyard. They were asked to imagine walking to the end of the path while remaining at the start position and indicate when they “reached” the end of the path. This was repeated on a 10m path. Participants were then timed as they actually walked the paths at the pace that they had just imagined. All times were measured by stopwatch.

To normalise the errors, the ratio: (*actual time–imagined time)/actual time* was calculated. Ratios greater than 0.2 (allowing a 20% error) were classified as over-estimates of walking speed and those less than -0.2 were classified as under-estimates.

#### Study 3—Perceived time to arrival of a moving object

Participants (N = 67) sat in a dimly lit large open laboratory and viewed an illuminated 10cm circle (a virtual ball projected from overhead) that moved across the laboratory floor (Fig 1B). It travelled with constant speed and direction towards a target at the end of a 2.4m path. For two-thirds of the path length the ball was in full view. It then disappeared into a virtual tunnel so that it was no longer visible for the remainder of its travel to the target. Participants indicated with a finger press at the time they thought the ball would reach with the target. Three trials each of six different speeds (1.15, 0.96, 0.77, 0.57, 0.38, 0.19ms^-1^) were presented in random order. Feedback on performance was not provided.

Control trials were made in which the ball was seen almost all the way to the target with the tunnel only long enough (10 cm) to hide the ball. Time errors of test trials were corrected by subtracting the mean errors of these control trials. The error in predicting the time of contact was measured (1ms resolution) using the software written to project the image (Labview 8.2; National Instruments, Texas).

### Ancillary physiological and cognitive tests

To seek functional correlates with the road-crossing behaviour and choices, participants undertook a range of physiological and cognitive tests that measured basic sensorimotor function, standing balance control, walking and stepping, timing and decision-making: (i) visual contrast sensitivity, measured by the Melbourne Edge Test, (ii) visual acuity measured at 3 m using a log MAR letter chart, (iii) simple reaction time, measured using a light as the stimulus and a finger-press as the response, (iv) quadriceps strength, measured isometrically in the dominant leg while seated, (v) postural sway when standing, measured using a simple sway recording device as the path excursion of the pelvis over 30 s while standing on a foam mat with eyes open, (vi) voluntary leaning balance control, measured by controlling the movement of the pelvis to track a narrow path that took the subject to the limits of balance [33], (vii) maximal reach was measured in the standing position [34], (viii) walking speed, the average speed while walking a 10 m path, (ix) stand and walk time, measured as the time taken to stand from sitting, walk three metres, turn and walk back to the chair and sit down (timed up and go test), (x) choice stepping reaction time, measured as the time to step onto an illuminated panel presented in random order as quickly as possible [35]. Cognitive function was measured using the Trail Making Tests A & B. Trails B—Trails A, attempts to remove the motor component of the test and is thus a purer measure of executive functioning.

### Self-reported behaviour—Everyday risk-taking scale

A 10-item self-report Everyday Risk-taking Scale was administered [36]. This questionnaire assessed risk-taking behaviour across a range of daily activities including pedestrian activities e.g. whether a person would cross against the lights or walk further to use a pedestrian crossing.

### Statistical analysis

The Mann-Whitney U or Kruskal-Wallis tests were used for non-parametric comparisons, and one-way and repeated-measures ANOVA with Student Newman-Keuls (SNK) correction for parametric comparisons. Variables with right-skewed distributions were log transformed for parametric analysis. Spearman’s rank correlation coefficients were used to examine the relationship between variables for non-parametric variables and Pearson’s correlation coefficients for parametric variables. Proportions were examined by χ^2^ test. Linear regression was used to describe associations between measures of time-to-contact and walking times. Behaviour with road crossing fell into five distinct categories: fail, ‘hit’, exact, safe and cautious. These groups were used in categorical analyses with measured physical and cognitive parameters. Absolute errors in the perceived time-to-arrival and walking speed tasks were used for associations with the five categories in the task crossing. *P*_α_<0.05 was taken to indicate statistical significance.

## Results

### Study 1—Road crossing

**Fig 2:**
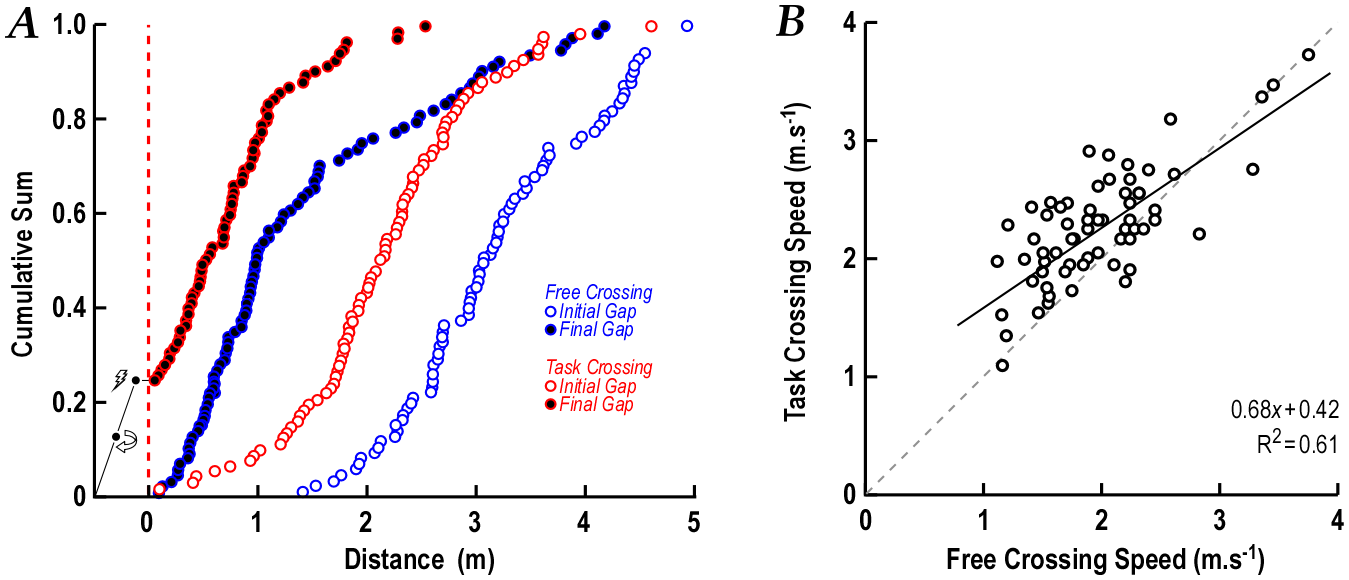
Initial and final gaps during the free crossing and task crossing. A. Initial and final gaps are presented as cumulative sum distributions for the group. Blue are the free-crossing data and red are the task-crossing data when given the additional ball-gathering task. During the task crossing, participants allowed smaller initial gaps leading some to be hit by the car and others missing the chance to cross (‘hit’, 9 participants; fail,11). B. Linear regression of walking speed during the task crossing by speed during the free crossing. Those who walked slowly during the free crossing increased speed more during the task crossing.

All participants understood and were able to carry out the required tasks. During the free crossing, all made a safe crossing. Fig 2A (blue points) shows the cumulative distributions of the initial and final gaps between the participant and car. The mean initial gap stepping onto the road was 3.3m±0.9 (±SD) and final gap was 1.5m±1.0. Some participants left small final gaps—two left less than 0.2 m—whereas some appeared overly safe and cautious. Women allowed larger final gaps than men (1.8m±0.2 and 1.2m±0.1, respectively, F_1,84_ = 9.3, *P* = 0.003 by ANOVA).

Undertaking the ball-gathering task while waiting to cross (task crossing) resulted in participants hurrying their crossing (Fig 2A, red points). The initial gap stepping onto the road decreased significantly (3.3m±0.9 to 2.2m±0.9; F_1,84_ = 119,*P*<0.001 by repeated measures ANOVA), and approximately one quarter of participants attempted unsafe crossings: 11% were ‘hit’ or veered away to outrun the car and 13% had to turn back and failed to cross. For successful crossings, the final gap was reduced significantly with the additional task (1.5m±1.0 to 0.7m±0.6; F_1,84_ = 50.0, *P*<0.001). Five sub-groups (fail (11 participants), hit (9), exact (14), safe (38), cautious (13)) were defined on the basis of performance in this task (see Fig 2). In the task crossing condition, there was no difference between the final crossing gaps of men and women (0.7m ± 0.9 and 0.8m ± 0.1 respectively, F_1,84_ = 0.4, *P* = 0.53).

Walking speed during the crossing significantly increased from 1.02ms^-1^±0.34 for the free crossing condition to 1.15ms^-1^±0.31 for the task crossing condition (F_1,84_ = 12.1, *P*<0.001 by repeated measures ANOVA). Fig 2A and 2B show that the slow walkers in the free crossing increased their speed the most whereas the faster walkers had similar speeds in both crossings.

**Fig 3:**
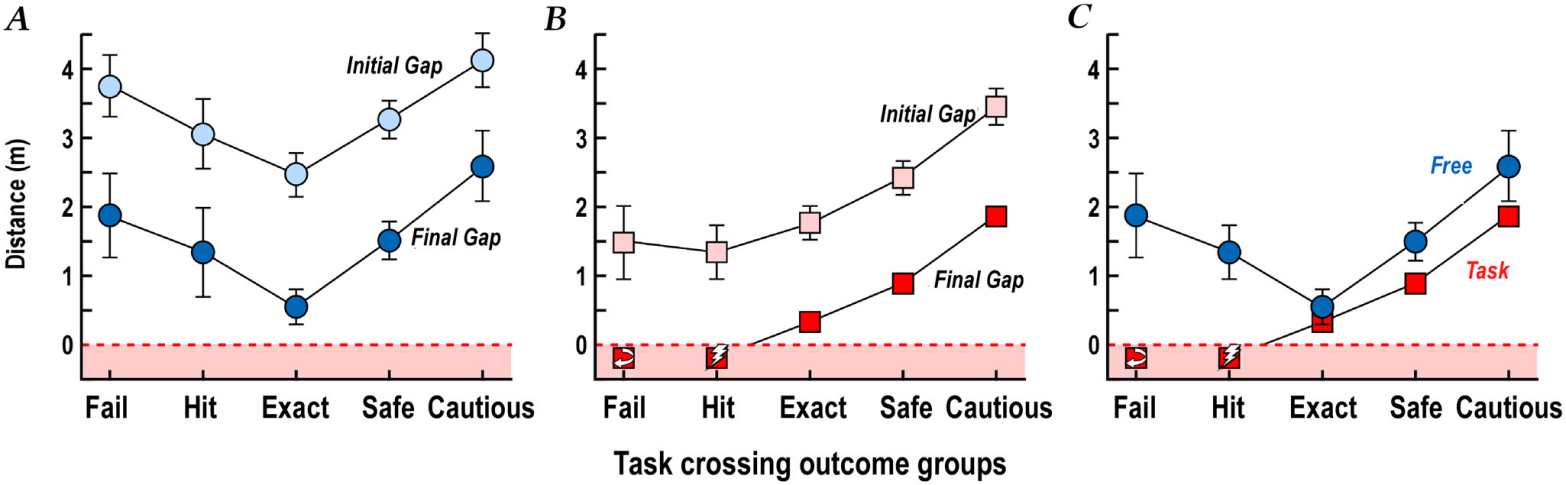
Initial and final gap distances by crossing group. A. Initial gap and final gap distances (mean ± SEM) for the five road-crossing groups are plotted for the free crossing. B. When participants had the additional task, smaller clearance distances were allowed by all groups. Those that were hit or failed in the task crossing left larger clearances in the free crossing. They behaved like those in the safe and cautious groups. C. Comparison of the final gap distances for the free and task crossings.

Fig 3 shows the comparison of the mean initial and final gaps for each of the task crossing outcome groups for both the free crossing and task crossing conditions. In the free crossing (Fig 3A), the *exact* group left the smallest initial gap before crossing whereas those in the *fail*, *hit*, *safe an*d *cautious* groups left larger gaps (F_1,84_ = 9.4, *P*<0.001; post-hoc *P* <0.05). Fig 3B shows the initial and final gaps of these groups during the task crossing. Compared with the free crossing, all groups left smaller initial gaps in the task crossing but the hit and fail groups changed their behaviour the most, resulting in a collision or failing to cross. The final gaps for the free and task crossings are compared in Fig 3C for the five task crossing outcome groups. Both the hit and fail groups had allowed large gaps in the free crossing.

In the ball-gathering task, 10±2 balls were gathered in 14.8±2.2s. Those who gathered more balls left smaller initial gaps in crossing (r = -0.33, *P*<0.01) but there was no effect on the final gap (r = -0.1, *P* = 0.38). The ball-gathering task was performed more slowly when attending to the crossing than when tested as a single task (0.7 balls.s^-1^±0.12 and 0.9 balls.s^-1^±0.16 respectively; F_1,84_ = 152, *P*<0.001 by repeated measures ANOVA).

### Study 2—Perceived time to walk to a target

Only one participant had difficulty understanding the imagined walk and was not tested. The remainder indicated that they understood and gave consistent responses. For example, all indicated a shorter time for the 5 m than the 10 m imagined walk (mean ratio 0.53±0.12).

**Fig 4:**
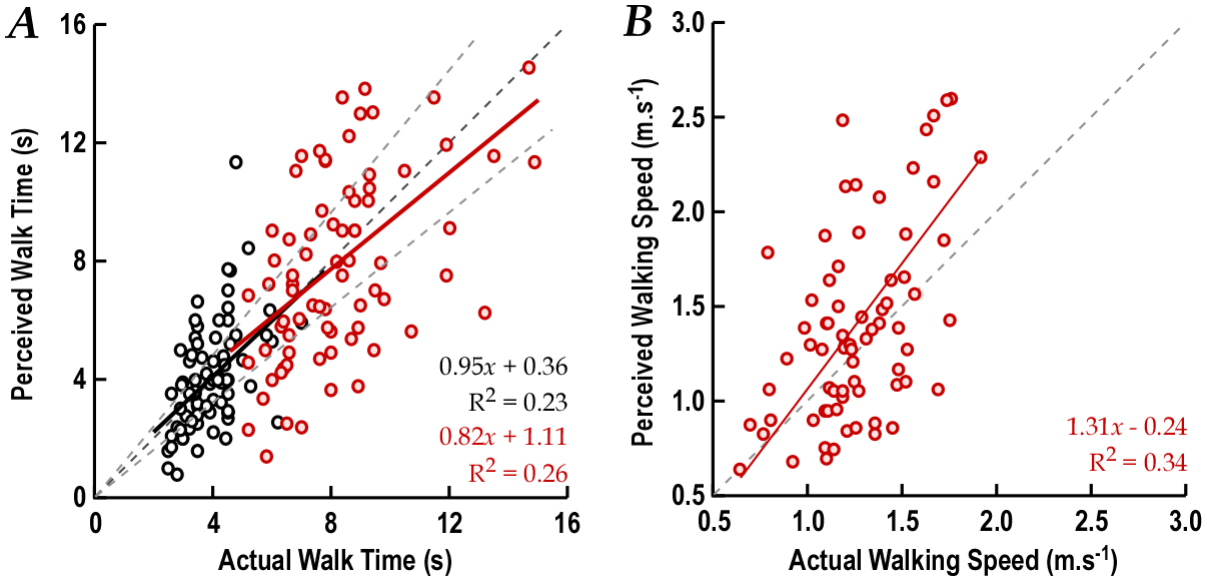
Perceived and actual walking times and speeds. A. Perceived walk time is plotted against actual time taken to walk the 5 m path (black) and 10m path (red). The equality and 20% error lines are shown. B. Perceived walking speed is plotted against actual speed (means of 5m and 10m path values). Participants tended to overestimate their walking speed.

Across participants, perceptions of time needed to walk the 5m and 10m paths showed strong relationships with the actual walk times (Fig 4A: overall β = 0.87, r^2^ = 0.49 by linear regression). There was however a wide range of relative perceptual errors. The perceived time was overestimated by more than 20% in one-quarter of the walks and underestimated by more than 20% in one-third. Men were more likely to overestimate their walking speed than women (F_1,73_ = 4.0, *P*<0.05 by ANOVA). Overall, participants tended to overestimate their walking speed by 21% (by mean differences).

There was a negative correlation (r = -0.37, *P* = 0.002), between actual walking speed and the initial gap chosen in the free crossing. That is, participants who walked slowly left larger initial-crossing gaps. However, there was no association between errors in perceived walking speed and initial gaps in the free crossing.

### Study 3—Perceived time to arrival of a moving object

**Fig 5:**
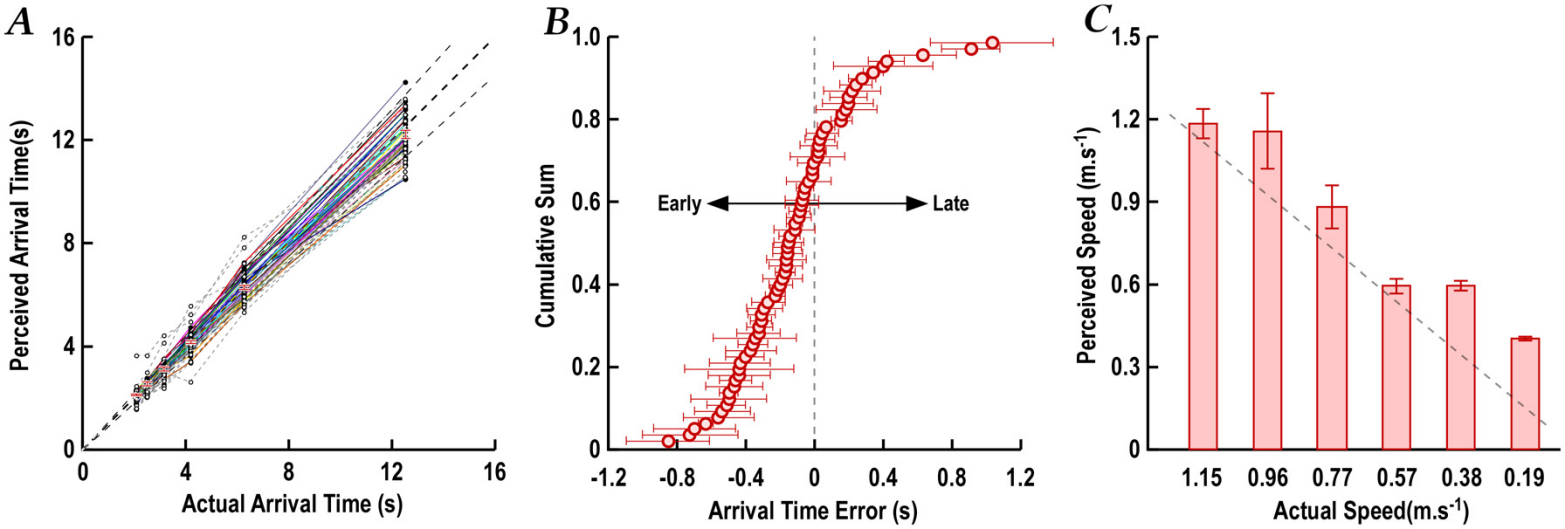
Perceived time to arrival and speeds of a moving object. A. Perceived arrival times are plotted against actual arrival times for each ball speed and participant. Black dots are the individual participant’s mean of 3 trials. The fanned parallel connecting lines indicate a high level of repeatability of relative perceived speeds across different ball speeds. Overlaid error bars are 95% confidence intervals of the means. The equality and 10% error lines are shown. B. The distribution of arrival time errors. Error bars represent the 95% CI of the mean value calculated across all trials for the participant. C. Mean (± SEM) perceived speed with each ball speed. The equality line indicates that participants overestimated the speed.

Fig 5A plots the responses of all participants for each of the six different speed profiles of the moving ball. After practice trials, all participants understood the task and gave reliable responses. This is shown by the proportional increase in the indicated time-to-arrival as the ball slowed.

The indicated time-to-arrival is approximately proportional for individual participants and extrapolates back to zero (Fig 5A and 5B), indicating that motor response times have been excluded by subtraction in the analysis and do not contribute to the errors observed. Participants tended to be consistent across ball speeds in underestimating or overestimating speed; *i*.*e*. if they overestimated at slow speeds, they also overestimated at high speeds, as reflected by the radial pattern for individual participants in Fig 5A and the absence of a significant within-subject effect of speed (χ^2^_25_ = 18.2, *P* = 0.82).

Overall, there was a greater tendency to underestimate than overestimate the time-to-arrival (Fig 5C), and this was greatest at slow speeds (83% underestimated at the slowest speed). In other words, participants acted as if they perceived the speed of the ball as greater than its actual speed.

To investigate the relationship between road crossing behaviour and estimated time to arrival of the moving ball, the ball speed that was closest to the car speed (0.57ms^-1^) was analysed. There was a positive correlation, albeit weak (r = 0.26, *P* = 0.05), between estimated time to arrival and the initial gap chosen for the free crossing. That is, participants who responded as if the ball was moving slower than actual left the largest crossing gaps.

There was a significant positive correlation between participants’ imagined and actual walking times with their estimations of arrival times of the moving object relative to actual (r = 0.4, *P<*0.001). This indicated that participants who estimated that they would take more time than they did when walking to a target also estimated that an external moving object would take more time than it did.

### Relationship between crossing outcomes and perceptual, physiological and cognitive measures

**Fig 6:**
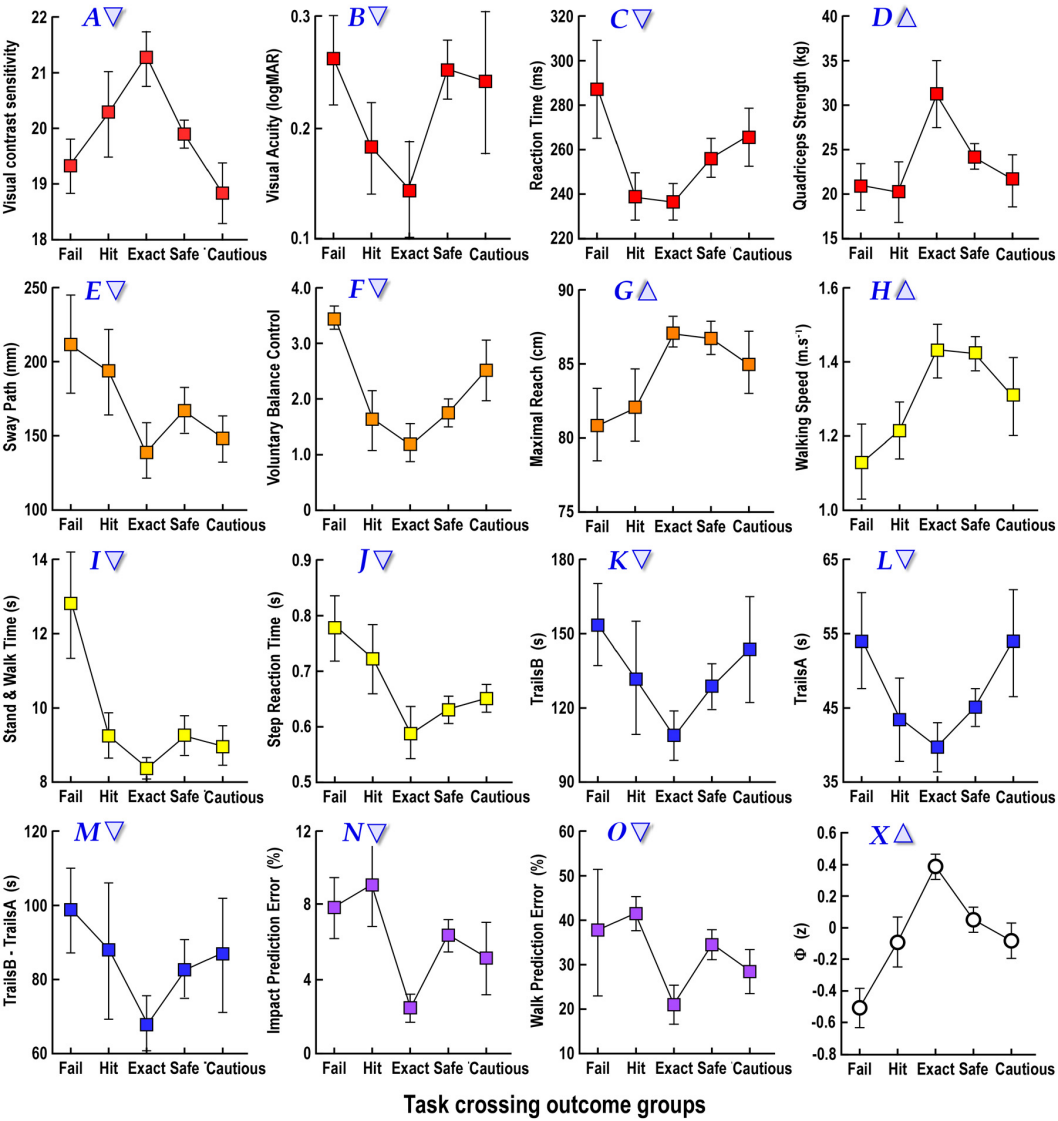
Physiological and cognitive associations with crossing behaviour in the task crossing. Mean performance (±SEM) in a range of physical and cognitive tests for the five groups classified according to road-crossing behaviour in the task crossing. For each graph, the triangle points in the direction of better performance. **A**,**B**,**C,D**. Tests of basic function (*visual contrast sensitivity*, *visual acuity*, *simple reaction time*, *strength*). **E**,**F,G**. Tests of standing balance control (*sway*, *voluntary balance control*, *maximal reach*). **H**,**I,J**. Tests of walking and stepping function (*walking speed*, *stand and walk time*, *choice stepping reaction time)*. **K,L,M.** Cognitive tests of search and set shifting (*TrailsB*, *TrailsA*, *TrailsB—TrailsA*). **N.** Arrival prediction error of the moving object. **O.** Arrival prediction error of the imagined walk. **X** shows the overall function score as a *z*-score average across all tests.

Performances in the physiological and cognitive tests were averaged for the five subgroups defined by crossing behaviour in the task crossing (see Fig 2; *fail*, *hit*, *exact*, *safe*, *cautious*). Fig 6A–6O plots the group mean (±SEM) for each measure with the triangle pointing in the direction of good performance. The group that made *exact* crossings, leaving very small gaps but still executing a safe crossing, performed the best in all 15 tests. This included performance in the two sub-studies; the perceived walking speed task and the perceived time-to-arrival of a moving object task (see Fig 6N and 6O). A measure of overall performance (Φ) was calculated by averaging the *z*-score performances on each test (Fig 6X). It is clear from the patterns of individual and averaged test results that performance fell away in either direction from the *exact* group who had the best performance. The *fail* group—those who did not cross because they allowed insufficient time and had to retreat—performed poorly and significantly worse than the exact and safe groups (post-hoc P < 0.05).

### Self-reported behaviour—Everyday risk-taking scale

**Fig 7:**
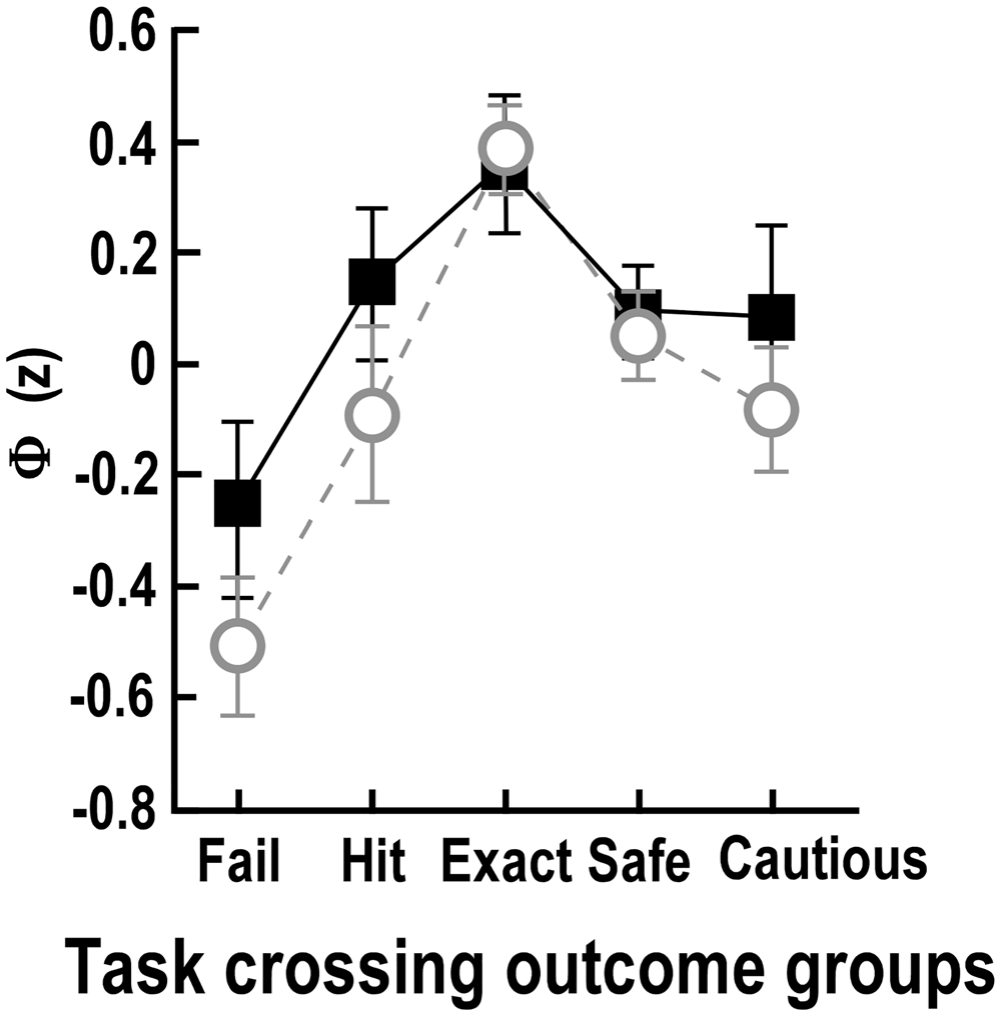
Everyday risk-taking scale scores by crossing outcome group. **A.** Scores on the Everyday risk-taking scale are plotted as filled squares for the five task-crossing outcome groups. A more positive score reflects more daring behaviour. Open grey circles show averaged normalised performances on tests of physical and cognitive function (reproduced from Fig 6X). A more positive score reflects better function. Scores are presented as means (z units ± SEM).

More risky behaviour, as indicated by the *Everyday risk-taking scale*, was associated with better overall function as measured by the aggregate physical-cognitive functions score, Φ, (r = 0.47, *P*<0.001). Fig 7 plots the mean score (±SEM) for each of the road crossing outcome groups. The *exact* group reported greater levels of risky behaviour and less caution than the other groups. These self-reported results show a remarkable similarity to the performance in the physical and cognitive tests for these groups (see Fig 7-reproduced from 6X). Thus, these self-reports appear to reflect objectively measured function rather than the risk taken in the road crossing tasks.

## Discussion

The main study aims were to determine the effect of an additional task on crossing decisions and to identify perceptual, physical and cognitive factors that contribute to safe and unsafe crossing behaviour. With respect to our hypotheses we found that: (i) participants who had accurate perceptions of the speed of moving objects and own gait speed were more likely to make good road-crossing decisions; (ii) participants with reduced physical and cognitive function made more unsafe crossing decisions and (iii) those who made unsafe crossing decisions reported cautious behaviour in everyday life. Importantly, the addition of the additional task was necessary to reveal crossing errors.

In the free crossing condition, all participants were able to cross the road safely. In the task crossing with the additional ball-gathering task, the group that made exact crossings did not change their behaviour from the free crossing. This indicates that this group could make precise judgements and execute them as well as attend to two tasks and prioritise their competing needs. For the other groups (fail, hit, safe and cautious), the additional task resulted in them allowing smaller clearances. Some, however, were still able to prioritise (*safe* and *cautious*) and cross successfully whereas others (*hit* and *fail*) did not prioritise and could not cross. The *hit* group appeared to either not reassess or to err in reassessing the crossing whereas the *fail* group appeared to have either correctly reassessed and retreated or were so distracted by the additional task that they missed the opportunity to cross completely.

In uncomplicated crossings of one-way roads, older people make fewer risky crossing decisions. They are able to judge vehicle approach appropriately and integrate this information with their own physical ability [25, 30]. Complex, multiple-lane crossings and high speed traffic pose the greatest threat to older people [21, 25, 29, 37]. However, even a “simple” road crossing can often be complicated by distractions and concurrent activities that demand attention; *e*.*g*. carrying items, conversation, looking for a bus, and using phones alter crossing behaviour [38] and affect gait [39, 40].

We performed two sub-studies to investigate factors that may contribute to unsafe crossing decisions. The first measured how accurately people estimate the time taken to walk to a target. Here we found that older people overestimated their walking speed. This result, previously shown by Dommes and colleagues [29], was shown to have no association with unsafe crossing decisions in a virtual crossing task. However, our results show that good judgment of walking speed was associated with exact crossing decisions in the task crossing condition. The second sub-study measured people’s ability to perceive the time to arrival of a moving object. Similar to the above result, good predictions in the time-to-arrival test was associated with exact crossing decisions in the task crossing, while poor performance was associated with unsafe crossing decisions. This finding supports previous research that has found distortions in time-to-arrival estimates are associated with risky crossing decisions in virtual crossing studies [29, 30].

In terms of physiological measures, poor sensory acuity could lead to coarse estimates of the time to arrival of oncoming vehicles and increase prediction errors. Further, performing a concurrent task requiring vision will result in task switching or the equivalent of “missing frames” (as in a vintage film with a slow frame rate) with regard to the observation of the approaching vehicle. A decision such as when to cross could therefore depend on just a few or even a single estimate. Indeed, it has been reported that older adults are less able to discriminate speed and behave in road crossing as if they respond more to the distance than the speed of an approaching vehicle [29, 37] which would occur if the decision was based on a brief image of the target.

The findings of this study agree with previous reports that older adults underestimate time-to-contact of moving objects compared with young adults [41–43]. It has been proposed that this reduces their risk of accidents [44]. However, contrary to this expectation, participants who overestimated (or perceived a late arrival of the moving object) left larger gaps in crossing. This suggests that predicting time-to-contact of the vehicle might not be crucial in road crossing calculations. This has also been suggested for driving performance in older people [45]. On average, older participants erred in perceiving an earlier arrival of a moving target than actual. Applying this to a collision avoidance model leads to an erroneous conclusion that older people would have less pedestrian accidents. However, participants also perceived that they would walk to a target in less time than actual. This suggests that estimation of one’s own mobility rather than the motion of the vehicle is the more critical factor. Although not significant due to small numbers of participants in the sub-group comparisons, it is worth noting that all the participants who were hit or fled to avoid being hit overestimated their walking speed in the 5 m walk. Systematic errors in predicting time-to-contact and time-to-walk form an appealing causal hypothesis to explain pedestrian accidents. However, the covariance with other sensorimotor functions and the size of these effects relative to discrepancies in crossing decisions suggests that these “perceptual impairments” may be indicators of general sensorimotor and cognitive impairments rather than a direct causal factor driving choices.

Poor cognition has been shown to predict unsafe crossing decisions in virtual-road crossings [26, 27, 29, 30] and there is an increased incidence of neurodegenerative pathological changes among older people killed in pedestrian accidents [46]. During the task crossing, participants had to divide their attention between two tasks—separate the balls and observe the car: discrete tasks that both rely on attention and working memory. Participants recruited for this study had no significant cognitive impairment based on a MMSE score of 24 or more (mean 28.5). However, the different behavioural groups performed differently in many tests of physical and cognitive function (see Fig 6). Overall, the group that made *exact* crossings in the task crossing had the best function in all tests of physical and cognitive function. Those who were hit or were cautious had poorer function, while those who failed to cross had the poorest function of all. While often not statistically significant in themselves, the accordance across the different domains suggests that these are real phenomena and argues for an underlying construct or mechanism.

It is possible that errors could arise through poor task performance and insufficient compensation for reduced ability. The concept of compensation prominent in ageing research is that when functional demands are high and when a person is unaware of how age has affected performance, the accumulation of age-related losses in many aspects of physical function overwhelms the ability to compensate [47–50]. However, the task used in the present study was not overwhelming. The less physically capable left longer gap distances in the free crossing, which could be interpreted as awareness of and compensation for a decline in physical function. Thus, because of this compensation, a simple model equating poor function with an increased risk of collision does not apply. In fact, the larger final gaps for these people indicate overcompensation, although this might be explained if they also allowed for greater performance variability.

Models related to pedestrian behaviour indicate the strong influence of individual characteristics on pedestrian flow and highlight the effect of maladaptive decisions, unexpected behaviour, or blindly following others [51, 52]. In our experiment, the car’s speed and path were fixed and the pedestrian was constrained to a fixed crossing path. However, the heterogeneous relationship between traffic and pedestrians may also play an important role in crossing outcomes for older people. In complex road crossing situations that involve multiple cars and bicycles travelling in opposite directions at varying speeds, and other pedestrians negotiating the traffic, there are likely other dangerous situations for older pedestrians. Future studies that include unexpected changes to the speed and trajectory of the car, common in every day crossing situations, would provide further insight into the individual characteristics and behaviour of older pedestrians that affect road safety [16]. Future developments of intelligent transport systems may also influence and improve road safety for older people [53, 54].

In completing the everyday risk-taking questionnaire, the exact group reported being the most daring while both the *hit* and *cautious* groups reported they avoided risk-taking. Thus, we found no evidence that crossing errors were the result of a “risk-taking” type [55, 56]. This finding is consistent with previous studies that showed older adults report more cautious pedestrian behaviour than younger adults [57, 58] although their actual crossing choices in a simulation study do not reflect this caution [22]. The results suggest that psychometric tests used to assess confidence and cautiousness such as the ABC [59] and *FES-I* [60], might not be appropriate for many situations because they are not referenced to individual ability [36], but instead population norms and expectations.

### Limitations

In the present study participants were only tested once so it is possible the cautious and risky ends of the spectrum are not fundamentally different. It is possible that the person who erred in the direction of apparent caution on one occasion might err with the wrong decision in the direction of daring on another occasion because of chance events in the decision process. Second, given the slow velocity of the car in these experiments, it is possible that these road-crossing judgements do not reflect real-life behaviour with much faster velocities. There is no doubt that participants allowed smaller time gaps than are generally observed in real situations and that being hit by this lightweight car at these speeds represents minimal hazard. Third, it is likely that the ball-gathering task assigned to participants here, was not as demanding and distracting as events and situations often encountered in real-life. Finally, it is acknowledged that the sample size was marginal for some of the statistical analyses performed, particularly when participants were categorised into sub-groups.

## Conclusions

In the simple crossing task, participants had no difficulty making safe crossings. However, when attention was divided, some participants made inappropriate and risky decisions leading to unsafe crossings. Across a wide range of physical and cognitive measures, which included estimating time-to-arrival and walking time, a consistent pattern was observed. Participants who made precise crossing decisions performed well in the perceptual, physical and cognitive tests and the precisions of their crossings were not significantly altered by the additional task. In contrast, participants who made unsafe and overly cautious decisions did not perform as well in the physical and cognitive tests. The precise crossers were more likely to report being daring in real life whereas the remainder more likely reported being cautious. Thus, it appears that self-reported risk taking behaviour does not reflect actual risk-taking behaviour but rather indicates good physical and cognitive function that enables accurate performance with low risk.

## Supporting Information

S1 File Study results. Spreadsheet containing results from the study.
(XLSX)
